# A Novel Three-Dimensional Stereoscopic Navigation System for Laparoscopic Anatomic Liver Resection

**DOI:** 10.7759/cureus.82214

**Published:** 2025-04-13

**Authors:** Kotaro Ito, Takao Ide, Tomokazu Tanaka, Hirokazu Noshiro

**Affiliations:** 1 Department of Surgery, Saga University, Saga, JPN

**Keywords:** 3d, blood loss, laparoscopic anatomic liver resection, navigation, three-dimensional, three-dimensional stereoscopic navigation

## Abstract

Introduction: Compared to viewing three-dimensional (3D) reconstructed images in a planar format, visualizing them in stereoscopic 3D using virtual reality technology enables intuitive and rapid recognition of spatial relationships. 3D stereoscopic navigation systems are expected to enhance the safety of liver resection by facilitating the precise localization of tumors and blood vessels. We recently introduced a novel 3D stereoscopic navigation system that allows real-time stereoscopic display of 3D simulations from any directional view selected by the attending surgeons during procedures. No reports have described the surgical advantages of the 3D stereoscopic navigation system in laparoscopic liver resection. In the present study, we retrospectively analyzed the usefulness of this novel 3D stereoscopic navigation system during laparoscopic anatomic liver resection (LALR).

Methods: Forty-four patients who underwent LALR from January 2021 to September 2023 were enrolled in this study. The patients were divided into two groups, each comprising 22 patients who underwent surgery with or without the current 3D stereoscopic navigation system. To determine the advantages of the navigation system, the perioperative surgical results and accuracy of the excised liver volume were compared between the two groups.

Results: The patients’ characteristics were not significantly different between the two groups. The estimated blood loss volume in the group with 3D stereoscopic navigation was significantly lower than that in the group without 3D stereoscopic navigation (118 vs. 218 mL, respectively; p = 0.048). There were no significant differences in the other surgical results between the two groups.

Conclusion: The use of this novel 3D stereoscopic navigation system reduces intraoperative blood loss and facilitates precise anatomical identification during LALR.

## Introduction

Anatomic liver resection is defined as complete resection of the liver parenchyma within the responsible portal venous region, and it is performed for the treatment of hepatic tumors [1]. Although laparoscopic liver resection has become more common, laparoscopic anatomic liver resection (LALR) remains a difficult and challenging surgical procedure due to the high risk of vascular injury [2,3].

Three-dimensional (3D) simulations based on contrast-enhanced computed tomography (CT) images provide more precise recognition of the anatomy and are commonly used in surgeries involving multiple regions. Especially in hepatic resection, because it is difficult to recognize intrahepatic anatomic structures such as blood vessels and tumors, implementation of 3D simulation to examine the anatomy is critically important to reduce intraoperative and postoperative complications during LALR.

When using a planar display, rotating 3D-reconstructed images is required to understand the spatial relationships, such as the anterior-posterior positioning of objects. In contrast, viewing these images in stereoscopic 3D allows for immediate and intuitive recognition without the need for rotation.

Various intraoperative navigation systems have been developed. A few reports have described 3D stereoscopic navigation systems that allow stereoscopic viewing of 3D simulations during liver surgery [4]. A novel 3D stereoscopic navigation system called Atrena (AMIN, Tokyo, Japan) was recently released. This system displays real-time 3D stereoscopic images of 3D simulations using virtual reality technology from any direction selected by the surgeon during surgery. No reports have focused on the surgical advantages of this real-time 3D stereoscopic navigation system in laparoscopic liver resection.

In this study, we retrospectively analyzed the advantages of this novel real-time 3D stereoscopic navigation system for LALR, focusing on short-term surgical outcomes and the accuracy of liver resection volume.

## Materials and methods

Patients

This study involved 44 consecutive patients who underwent LALR for the treatment of hepatic tumors from January 2021 to September 2023 at Saga University Hospital, Saga, Japan. Indications for LALR were as follows: 1) age over 18 years old, 2) Child-Pugh class A or B in liver function, 3) hepatic tumors detected by CT and/or magnetic resonance imaging, 4) the number of hepatic tumors less than three, 5) no extrahepatic metastases, and 6) existence of good pulmonary function tolerant to the pneumoperitoneum. The following exclusion criteria were used: 1) urgent surgery, 2) nonanatomical hepatectomy, and 3) simultaneous surgery on other organs.

We started using 3D stereoscopic navigation in December 2021, and since then, 3D stereoscopic navigation has been used in all cases of LALR. In this study, the study period was set so that the number of cases before and after the introduction of 3D stereoscopic navigation was equal. Two groups were analyzed: those who underwent LALR with the 3D stereoscopic navigation system and those without it. All LALR procedures were performed by three trained operators, each with over 15 years of surgical experience and more than 50 laparoscopic liver resections. This study was approved by the Ethics Committee of Saga University Hospital (2019-09-R-03). Written informed consent was obtained from all patients.

Creation of 3D images

Three-phase contrast-enhanced CT images in the arterial, portal, and venous phases were taken at the appropriate time by bolus tracking. Before surgery, CT images were reconstructed using Ziostation2 software (Ziosoft, Newark, CA) to create a 3D simulation, which helped identify the locations of blood vessels and tumors, determine hepatic resection lines, and estimate the volume of liver to be excised.

Using the Ziostation2 software, a 3D simulation was created from CT images by the operator. When the simulation data were transferred to Atrena, a 3D stereo navigation system, the software automatically generated a 3D stereoscopic image. Therefore, minimal additional time was required to create the 3D stereoscopic image for Atrena.

Preoperative examinations and preparations

Indocyanine green (ICG) (0.5 mg/body) was administered intravenously one to three days before surgery, and the ICG concentration in the blood was measured 15 minutes later to evaluate liver function. In addition, ICG administered preoperatively was used with fluorescence observation to distinguish between the tumor and normal liver parenchyma during surgery [5].

Surgery

**Figure 1:**
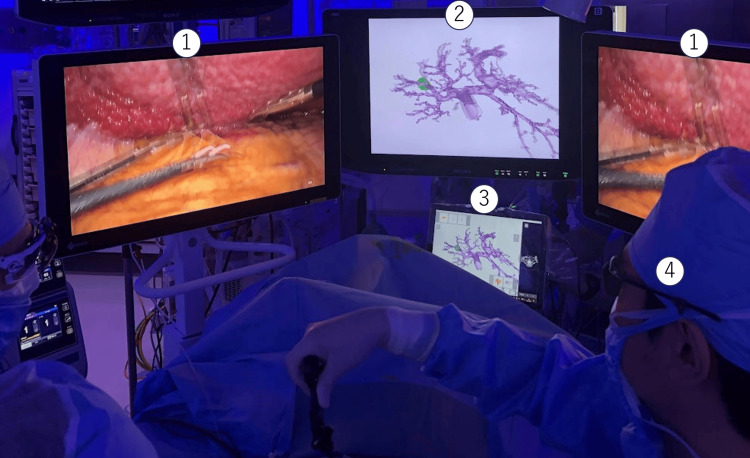
Intraoperative scene and intraoperative placement of equipment 1: 3D monitor for endoscopy; 2: 3D monitor for 3D stereoscopic navigation system; 3: operating device for 3D stereoscopic navigation system; 4: polarized glasses.

The operating device for intraoperative 3D stereoscopic navigation was managed in a sterile field. The display system for 3D stereoscopic navigation was suspended from a ceiling pendant and set near the endoscopic monitor for the main surgeon (Figure 1, Video 1). The 3D stereoscopic navigation system was utilized to verify anatomical structures and the dissection plane of hepatectomy, often during the release time of Pringle maneuver. In surgeries without the 3D stereoscopic navigation system, a fixed planar image of a representative 3D simulation was displayed on the suspended monitor. Surgeons were required to wear polarized glasses to view the navigation images in stereoscopic vision. However, since they were already wearing glasses to view the endoscopic images, there was no additional burden on the surgeons. The 3D stereoscopic navigation system was intuitive to operate and could be fully utilized from the outset of the procedure.

**Video 1:** Surgical video of the use of 3D navigation Surgery was performed in a specialized room for endoscopic surgery. All room lights were dimmed except those illuminating the anesthesiologist’s table; the remaining lights were enhanced with a blue tint during the procedures to obtain better visualization of the real endoscopic images. Three 3D monitors for the individual surgeons were suspended from the ceiling and freely set to obtain an accurate 3D image provided by the 3D endoscopic surgical system 3D: three-dimensional

The surgical procedures for LALR were carried out by isolating the targeted Glissonean pedicle at the hilum, with appropriate transection planes built sequentially according to anatomic landmarks, as we previously described [6]. When the target Glissonean pedicle was far from the hilum, such as in subsegmentectomy of segment 8, the Glissonean pedicle was occasionally isolated while dissecting the liver parenchyma. Intraoperative identification of anatomical boundaries is essential for clear margins. After ligation of the targeted pedicle, ICG was intravenously administered, and hepatic division was performed according to the negative staining method on the overlaid infrared fluorescence 3D image.

Accuracy of liver excised volume

The surgeon measured the estimated volume based on a 3D simulation created using the Ziostation2 software, and the anatomical location of the portal vein and hepatic vein determined the extent of resection. The estimated volume of the excised liver (milliliters) was corrected by the standardized liver density (1.05 g/mL) as follows: Estimated volume of excised liver (mL) = actual weight of excised liver (g) / liver density (1.05 g/mL) [7]. The expected excised volume was calculated using the 3D simulation program. The accuracy of the expected and actual volumes of the excised liver was determined using the index of the accuracy of the excised liver volume (expected excised volume / estimated excised volume).

Statistical analysis

The following clinical characteristics were retrospectively obtained from a prospectively maintained comprehensive database and the patients’ medical records: age, sex, body mass index, performance status, disease, Child-Pugh classification, ICG excretion rate, tumor size, surgical procedure, operative time, intraoperative blood loss, hospital stay, postoperative complications, and 90-day mortality. Postoperative bile leakage was defined as grade B or higher according to the International Study Group of Liver Surgery classification [8]. Patients’ characteristics are expressed as median and interquartile range. Student’s t-test, Pearson’s chi-square test, Fisher’s exact test, or Wilcoxon’s test were used to analyze categorical parameters, as appropriate. Correlation for the accuracy of the excised liver volume was determined using Pearson’s test. A p value of <0.05 was considered statistically significant. All statistical analyses were performed using JMP Pro version 16 (SAS Institute, Inc., Cary, NC).

## Results

Patients’ characteristics and surgical results

The characteristics of the 44 patients and the surgical results are summarized in Tables 1, 2. No significant differences in the patients’ characteristics were observed between the groups with and without use of the 3D stereoscopic navigation system (Table 1). There was no difference in the number of cases among surgeons between the two groups. No patients in either group were converted to any type of laparotomy. The mean operating time was not significantly different between the two groups with, 306 mL (range, 253-444 mL), and without, 322 mL (range, 213-419 mL), the use of the 3D stereoscopic navigation system (p = 0.655). However, the median intraoperative blood loss volume was significantly lower in the group with, 118 mL (range, 48-252 mL), than without, 218 (range, 113-526 mL), the use of the 3D stereoscopic navigation system (p = 0.048). Postoperative Clavien-Dindo grade ≥3 complications were observed in three patients in each group. These complications included two cases of bile leakage in each group and one case of postoperative paralytic ileus in the group with the use of 3D stereoscopic navigation. One patient in the group without the use of 3D stereoscopic navigation died of postoperative toxic epidermal necrolysis. The median postoperative stay was nine days (range, 8-13 days) in the group with use of the 3D stereoscopic navigation system and 10 days (range, 8-12 days) in the other group. There were no significant differences in the postoperative complication rate, the rate of a cancer-positive surgical margin, or the length of postoperative stay between the two groups (Table 2).

**Table 1:** Characteristics of patients who underwent LALR with versus without 3D navigation 3D: three-dimensional; LALR: laparoscopic anatomic liver resection; BMI: body mass index; HCC: hepatocellular carcinoma; ICC: intrahepatic cholangiocarcinoma; ICG R15: indocyanine green retention rate at 15 minutes

Characteristics of patients	With 3D stereoscopic navigation (n = 22)	Without 3D stereoscopic navigation (n = 22)	p value	Chi-square value
Age (years)	71 (57-77)	74 (65-81)	0.320	-
Gender, n (%)				
Female	４ (18.2%)	7 (31.8%)	0.293	1.102
Male	18 (81.8%)	15 (68.2%)		
BMI	22.7 (20.3-24.9)	21.7 (19.8-24.3)	0.438	-
Performance status, n (%)				
0	16 (72.7%)	18 (81.8%)	0.470	0.520
1	6 (27.3%)	4 (18.2%)		
Diagnosis, n (%)				
HCC	13 (59.1%)	15 (68.2%)	0.316	3.537
ICC	1 (4.6%)	2 (9.1%)		
Metastasis	3 (13.6%)	4 (18.2%)		
Other	5 (22.7%)	1 (4.5%)		
Child-Pugh classification, n (%)				
A	21 (95.5%)	22 (100%)	0.235	1.410
B	1 (4.5%)	0 (0%)		
ICG R15, %	13.2 (9.6-23.5)	16.5 (9.3-24.5)	0.445	-

**Table 2:** Surgical results 3D: three-dimensional

Surgical result	With 3D stereoscopic navigation (n = 22)	Without 3D stereoscopic navigation (n = 22)	p value	Chi-square value
Procedure, n (%)				
Hemihepatectomy	2 (9.1%)	4 (18.2%)	0.187	3.348
Sectionectomy	14 (63.6%)	8 (36.4%)		
Segmentectomy	6 (27.3%)	10 (45.4%)		
Size of tumor, cm	28 (22-53)	28 (22-49)	0.650	-
Operation time, minutes	306 (253-444)	322 (213-419)	0.655	-
Blood loss, mL	118 (48-252)	218 (113-526)	0.048	-
Transfusion, n (%)	1 (4.5%)	1 (4.5%)	1.000	-
Conversion to laparotomy, n (%)	0	0	1.000	-
Postoperative complication, n (%)	3 (13.6%)	3 (13.6%)	0.634	-
Postoperative bile leakage, n (%)	2 (9.1%)	2 (9.1%)	1.000	-
Surgical margin, mm	3 (1-15)	4 (1-21)	0.776	-
90-day mortality, n (%)	0	1 (4.5%)	0.235	-
Postoperative stay, days	9 (8-13)	10 (8-12)	0.943	-

Validation of expected and actual excised liver volume

**Figure 2:**
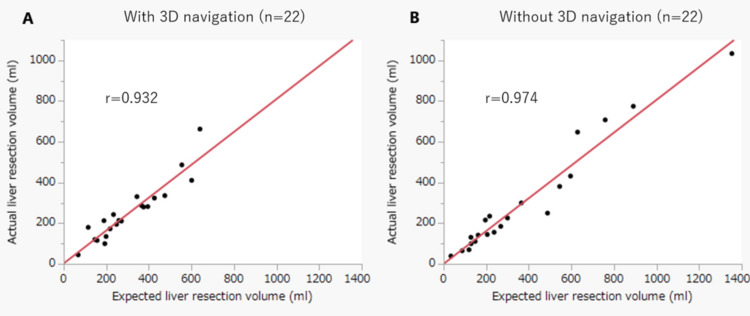
Relationship between expected and actual liver resection volume via Pearson’s test. (A) With 3D navigation. (B) Without 3D navigation 3D: three-dimensional

Validation of the LALR accuracy is shown in Table 3. The expected excised volume, estimated excised volume, and index of the accuracy of the excised volume showed no significant differences between the two groups. The proportions of the expected and estimated excised liver volumes showed significantly high correlations in both groups (Figure 2).

**Table 3:** Validation of LALR accuracy LALR: laparoscopic anatomic liver resection; 3D: three-dimensional

Hepatic resection volume	With 3D stereoscopic navigation (n = 22)	Without 3D stereoscopic navigation (n = 22)	p value
Expected excised volume, mL	253 (181-402)	226 (128-557)	0.953
Estimated excised volume, mL	224 (105-455)	149 (79-406)	0.980
Index of accuracy of excised liver volume	0.781 (0.701-0.898)	0.758 (0.696-0.955)	0.878

## Discussion

The hepatic vasculature and biliary system play a pivotal role in liver resection, where the precise identification and management of the hepatic vessels are crucial to ensuring adequate tissue perfusion and minimizing the risk of hemorrhage. The major components of the hepatic vascular system include the hepatic artery, portal vein, and hepatic veins. Understanding the distinct anatomical features of these vessels is essential for performing safe and precise hepatic resection procedures. In liver resection, it is crucial to either carefully preserve or safely divide both the vascular and biliary structures, depending on the segment being resected, to prevent complications and ensure optimal outcomes. Some liver tumors exhibit intravascular invasion, which facilitates the spread of metastases along blood vessels. In such cases, anatomical liver resection along the vascular structures can be a useful approach to ensure the complete resection of these metastases. In hepatobiliary surgery, numerous efforts have been made to enhance the recognition of hepatic vascular anatomy, including using 3D imaging simulations and surgical techniques, which have garnered significant clinical interest.

Because LALR remains a challenging surgical procedure, new advances in surgical techniques, including surgical devices, are needed to perform LALR more safely. In this study, we showed high accuracy of liver excision and significant reduction of intraoperative blood loss with the use of a novel 3D stereoscopic navigation system during LALR. This is the first report addressing the surgical benefits of the 3D stereoscopic navigation system in laparoscopic liver resection. Improved anatomic recognition using 3D stereoscopic navigation prevented unnecessary vessel injury and maintained an accurate orientation for liver dissection. The introduction of 3D stereoscopic navigation can be beneficial for ensuring the safety of laparoscopic liver resection; however, consideration should be given to the increased economic costs to the hospital associated with the implementation of software and display devices. From a cost-benefit analysis perspective, the initial investment in the system may be offset by improvements in surgical precision, reduced operative time, and potentially enhanced patient outcomes, making it a cost-effective tool in the long term.

This 3D stereoscopic navigation system, called Atrena, differs from previous navigation systems in that it does not display 3D simulations as 2D flat images but presents 3D simulations as stereoscopic images, enabling the user to intuitively and quickly recognize depth and spatial relationships without the need to rotate the image. In addition, it can be used in real time on the operation table by the attendant surgeon themselves, who can freely select the direction in which to view the anatomical structures. Although several reports have described the use of Atrena in nephrectomy and pulmonary surgery [9,10], the use of Atrena in gastrointestinal and laparoscopic surgery has not been reported to date.

The improved anatomic recognition provided by 3D stereoscopic navigation may contribute to safer surgical procedures in difficult cases involving patients with large tumors or anatomic anomalies that make intraoperative anatomic recognition difficult. Some previous studies showed that the use of simulation reduced the operative time for liver resection [11,12]. However, no difference in the operation time was observed in the present study. Although there was no significant difference in resection margins in this study, improved anatomic recognition may also improve resection margins.

Atrena provides a real-time intraoperative stereoscopic display of 3D simulations at any angle under the surgeon’s control in a sterile field. It is a device specialized for surgery and can be easily operated by the surgeon or assistant, who simply touches the screen of the tablet device for operation. Compared with other 3D stereoscopic navigation systems, Atrena is easier and more convenient to operate during surgery. The surgeon and assistant can rotate, zoom, and move the 3D simulation by manipulating the tablet screen in real time during surgery. Notably, individual blood vessels and organs can be made translucent or hidden to improve recognition of the positional relationship between vessels and organs. Hepatic vascular anatomy can exhibit variations, and 3D stereoscopic navigation can reduce intraoperative vascular injury by enhancing anatomical recognition. Furthermore, volumetric measurements of the dominant hepatic region of the portal vein can be made intraoperatively on Atrena. By sharing 3D simulations intraoperatively among the surgical team, misunderstandings regarding the anatomy and the next surgical procedure can be avoided.

Accurate recognition of the anatomy is necessary for many gastrointestinal surgeries beyond liver resection, and 3D stereoscopic navigation is expected to be used for various minimally invasive gastrointestinal surgeries in the future. This novel 3D stereoscopic navigation system can also be applied in robotic surgery. Considering the advantages of its stereoscopic display, 3D stereoscopic navigation would be highly advantageous, especially in robotic surgery. 3D stereoscopic navigation is also useful as an educational tool because it can improve anatomic recognition by young surgeons [13].

However, it must be remembered that 3D simulation using 3D stereoscopic navigation is dependent on the quality of the contrast CT images, and poor quality may lead to misidentification [14]. Therefore, to create high-quality 3D simulations, it is necessary to adjust the concentration and speed of the contrast agent and to use techniques such as the bolus tracking method for appropriate CT images. The ability to create 3D simulations in reconstruction software needs to be improved.

This study had three main limitations: historical bias; a retrospective, single-center design; and a small sample size. The first case of laparoscopic liver resection in our institution was performed in 2009, and cases from January 2021 onward were analyzed in this study. Even in the group that did not use 3D navigation, the impact of historical bias was relatively small, as the procedure was no longer in the induction phase, and there was sufficient proficiency. Further prospective studies are needed to accumulate more cases.

## Conclusions

Our results revealed that LALR with the current real-time 3D stereoscopic navigation system significantly reduced blood loss. We consider that improvement of anatomic recognition facilitated by convenient real-time 3D stereoscopic navigation will further increase the quality of surgery.
